# A Smoking Prevention Program Delivered by Medical Students to Secondary Schools in Brazil Called “Education Against Tobacco”: Randomized Controlled Trial

**DOI:** 10.2196/12854

**Published:** 2019-02-21

**Authors:** Oscar Campos Lisboa, Breno Bernardes-Souza, Luiz Eduardo De Freitas Xavier, Matheus Rocha Almeida, Paulo César Rodrigues Pinto Corrêa, Titus Josef Brinker

**Affiliations:** 1 School of Medicine Federal University of Ouro Preto Ouro Preto Brazil; 2 National Center for Tumor Diseases German Cancer Research Center University of Heidelberg Heidelberg Germany; 3 Department of Dermatology University Hospital Heidelberg Heidelberg Germany; 4 German Cancer Consortium University of Heidelberg Heidelberg Germany

**Keywords:** smoking, tobacco, prevention, medical students, schools

## Abstract

**Background:**

Smoking is the largest preventable cause of mortality in Brazil. Education Against Tobacco (EAT) is a network of more than 3500 medical students and physicians across 14 countries who volunteer for school-based smoking prevention programs. EAT educates 50,000 adolescents per year in the classroom setting. A recent quasi-experimental study conducted in Germany showed that EAT had significant short-term smoking cessation effects among adolescents aged 11 to 15 years.

**Objective:**

The aim is to measure the long-term effectiveness of the most recent version of the EAT curriculum in Brazil.

**Methods:**

A randomized controlled trial was conducted among 2348 adolescents aged 12 to 21 years (grades 7-11) at public secondary schools in Brazil. The prospective experimental design included measurements at baseline and at 6 and 12 months postintervention. The study groups comprised randomized classes receiving the standardized EAT intervention (90 minutes of mentoring in a classroom setting) and control classes in the same schools (no intervention). Data were collected on smoking status, gender, social aspects, and predictors of smoking. The primary endpoint was the difference in the change in smoking prevalence between the intervention group and the control group at 12-month follow-up.

**Results:**

From baseline to 12 months, the smoking prevalence increased from 11.0% to 20.9% in the control group and from 14.1% to 15.6% in the intervention group. This difference was statistically significant (*P*<.01). The effects were smaller for females (control 12.4% to 18.8% vs intervention 13.1% to 14.6%) than for males (control 9.1% to 23.6% vs intervention 15.3% to 16.8%). Increased quitting rates and prevented onset were responsible for the intervention effects. The differences in change in smoking prevalence from baseline to 12 months between the intervention and control groups were increased in students with low school performance.

**Conclusions:**

To our knowledge, this is the first randomized trial on school-based tobacco prevention in Brazil that shows significant long-term favorable effects. The EAT program encourages quitting and prevents smoking onset, especially among males and students with low educational background.

**Trial Registration:**

ClinicalTrials.gov NCT02725021; https://clinicaltrials.gov/ct2/show/NCT02725021

**International Registered Report Identifier (IRRID):**

RR2-10.2196/resprot.7134

## Introduction

In 2015, smoking accounted for 156,216 deaths and 3.72 million disability-adjusted life years in Brazil, representing a direct cost for the health system of R$39.4 billion [1]. More than 30.0% of Brazilian boys and 27% of girls aged 13 to 15 years had tried smoking before the age of 12 [2]. Given the time adolescents spend in the school setting, schools represent an excellent opportunity to deliver smoking prevention programs.

### Current Knowledge on School-Based Tobacco Prevention

Most school-based tobacco control programs are ineffective, but data from Brazil remain scarce [3-5]. Recent trials on tobacco prevention in the school setting have focused on including school teachers in the intervention [6-8], with others involving families [9,10]. However, these studies concluded that the students’ environment (ie, peer group as well as parental behavior and school policies) plays a role in smoking initiation in adolescence.

A randomized controlled trial involving different school-based interventions to reduce the use of various psychotropic substances among 1316 students in Brazil showed mixed effects for different drugs; however, the study design had limitations that precluded interpretation [11]. Another study from Brazil analyzed the effectiveness of an educational intervention by the Brazilian Cancer Institute (INCA) on smoking among school adolescents. Those researchers randomized 32 schools to either control (no intervention) or intervention arms, with a total sample of 2200 students in grades 7 and 8 (aged 13-14 years). INCA members lectured teachers from schools in the intervention arm about tobacco control, with the expectation that those teachers would discuss tobacco-related topics with their students. No change in smoking prevalence was found at the study endpoint, but knowledge about passive smoking had improved [12].

### Education Against Tobacco

Education Against Tobacco (EAT) is a network of volunteer medical students and physicians from more than 80 medical schools in 14 countries worldwide that was founded in Germany in 2012 [13]. The network has its roots in school-based interventions delivered by medical students. These interventions cost approximately US $20 per participating class and can reach up to 50,000 students per year worldwide. EAT is also involved in medical education research on smoking cessation counseling, science-based multilanguage apps, and public awareness and advocacy for tobacco control [14-16].

A German quasi-experimental study showed the school-based intervention resulted in a significant reduction in smoking prevalence among secondary school students at 6-month follow-up [17,18]. A randomized follow-up study in Germany indicated effectiveness at the 12-month follow-up; however, the results were not significant because of a large loss-to-follow-up effect [19]. Recent studies indicate that physicians substantially undertreat tobacco addiction compared with other chronic conditions, such as diabetes or hypertension [20-22]. The school-based smoking prevention provided by EAT is thought to sensitize medical students for tobacco control in general, as well as having a direct effect on adolescents [13].

A facial-aging app was implemented as part of the school-based EAT interventions to increase effectiveness. Facial-aging interventions, in which a selfie is altered to predict future appearance, provide motivation for healthier behavioral choices in adiposity prevention, skin cancer prevention, and smoking cessation [14,16,19,23-39]. An explanation for these preliminary results may be the high importance of appearance for a persons’ self-concept, especially in adolescence [40]. However, to our knowledge, the only completed randomized trial that investigated the prospective effectiveness of a facial-aging intervention on actual behavior (smoking) was conducted by Burford et al [41]. In that study, five of 80 control group participants (6.3%) suggested they had quit smoking at the 6-month follow-up, and 22 of 80 intervention group participants (27.5%) reported quitting (*P*<.05).

This study aimed to determine the long-term effectiveness of the school-based EAT intervention in reducing smoking prevalence among secondary school students in Brazil, as per the study protocol [42].

## Methods

**Figure 1:**
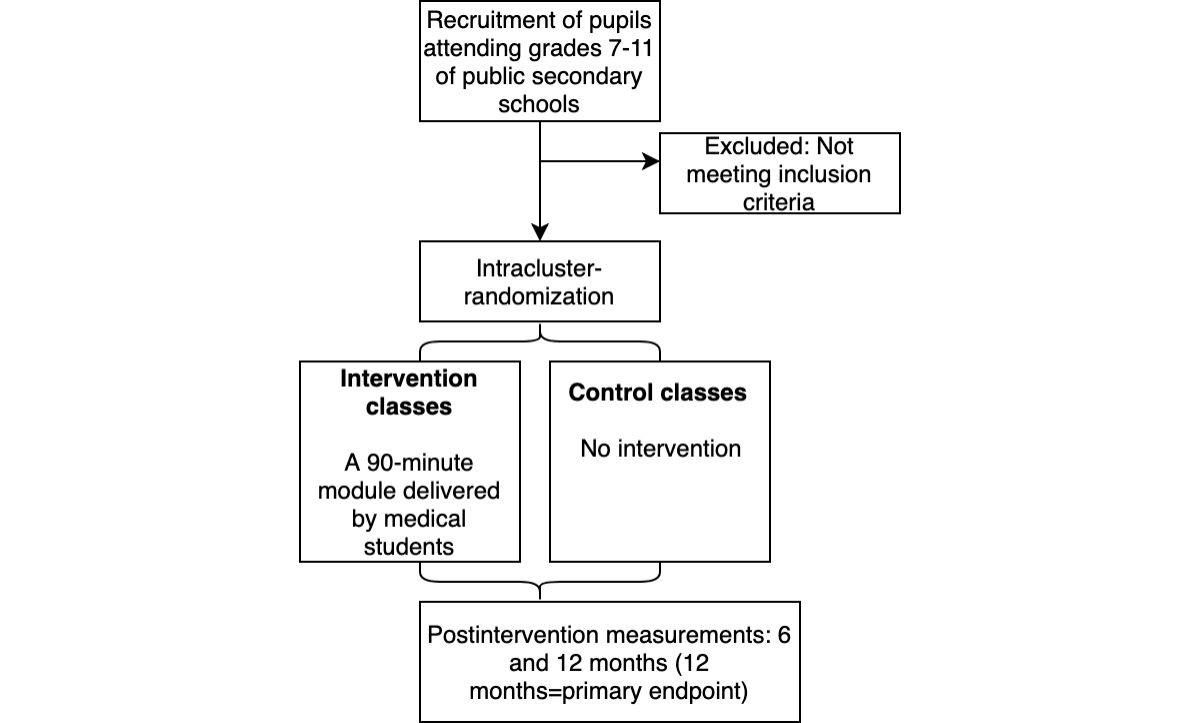
Study design and flow of participants.

A randomized controlled trial was conducted among 2384 adolescents in grades 7 to 11 from secondary schools in Brazil from February 2017 to June 2018 (Figure 1). All predefined time points were met. Details of the study design and the development of the questionnaire are outlined in our previously published study protocol [42].

### Participants

Students in grades 7 to 11 at secondary public schools were eligible to participate in this study. In total, 2348 secondary school students from 110 classes (from 14 eligible schools), who fulfilled the inclusion criteria, entered baseline data. Baseline data (t1) were collected from February 2017 to May 2017. Follow-up data (t2 and t3) were collected 6 and 12 months after that (from August 2017 to June 2018). Overall, 1353 participants provided data at both t1 and t3, which were used for primary endpoint analyses. The loss to follow-up effect was 42.38% (995/2348).

### Attrition Analysis

To evaluate attrition bias, participants who dropped out at follow-up (t3) were analyzed using logistic regression analysis (1=dropout and 0=analysis sample; t1 and t3 participation as the dependent variable). There was no systematic bias regarding the main effect of study group (intervention vs control; *P*=.24) and the interactions between study group and gender (*P*=.61), study group and age (*P*=.56), or study group and grade (grades 7-11; *P*=.10). However, there was systematic bias regarding the interaction of study group (0=control and 1=intervention) and smoking status (0=no tobacco consumption during the last 30 days and 1=at least one regular or straw cigarette, water pipe/hookah, or e-cigarette; odds ratio [OR] 0.63, 95% CI 0.42-0.94, *P*=.02). Among smokers, there were fewer dropouts in the intervention group than in the control group. For nonsmokers, the dropout rates were about the same in both study groups (intervention group/smoker: 52.1%, 114/219 dropout; control group/smoker: 63.4%, 116/183 dropout; intervention group/nonsmoker: 39.0%, 409/1084 dropout; control group/nonsmoker: 39.6%, 356/898 dropout).

The dropout probability differed for the main effects of four characteristics: smoking status (OR 2.67, 95% CI 1.95-3.66, *P*<.001), gender (OR 1.57, 95% CI 1.24-2.00, *P*<.001), age (OR 1.14, 95% CI 1.09-1.19, *P*<.001), and academic performance (OR 1.39, 95% CI 1.27-1.53, *P*<.001). Age and academic performance were used as the metric. The ORs represented a change in the characteristic by one level.

Dropouts distorted the remaining sample for the analysis in four directions: younger age, more girls, students with better performance, and fewer smokers. Reasons for loss to follow-up included identifier code not assignable, change of school, unauthorized absence from class (truancy), illness, or grade retention.

### Intervention: Education Against Tobacco

The EAT school-based intervention comprises a 90-minute module in the classroom setting (about 25 students per class) delivered by two medical students per classroom. The intervention was implemented by 36 volunteering medical students from the Federal University of Ouro Preto EAT group who received standardized training in advance, which was monitored via a performance questionnaire by the instructor. The program covered features of smoking that students can relate to in their everyday life in a gain-framed and interactive manner, which also involved a three-dimensional facial-aging app, “Smokerface,” developed by EAT. The intervention is described in detail in our study protocol [42].

### Outcomes

The primary outcome was the difference in smoking prevalence from baseline (t1) to 12 months of follow-up (t3) in the control group versus the difference from t1 to t3 in the intervention group. The differences in smoking behavior (smoking onset, quit attempts) between the two groups were studied as secondary outcomes, along with gender-specific effects.

### Data Entry

Data entry was performed manually at the Federal University of Ouro Preto in Brazil, using Microsoft Excel (Microsoft Corp, Redmond, WA, USA) and SPSS (IBM Corp, Armonk, NY, USA).

### Statistical Analyses

We used chi-square tests, *t* tests, and Fisher exact tests to examine baseline differences. The effects of predictors (gender and social characteristics) on smoking behavior at 12 months were calculated using robust panel logistic regression analysis. The significance level was set at 5% for *t* tests (two-sided) with 95% CIs (two-sided). Statistical analyses were performed using SPSS version 23 (IBM Corp, Armonk, NY, USA). The group allocation of the study sample was based on class level. Statistically robust panel logistic regression was used (SPSS GENLINMIXED procedure) to account for clustering. This procedure was also used to calculate the difference in the smoking prevalence from baseline to 12-month follow-up in the control group versus that in the intervention group (primary outcome). The number needed to treat (NNT) was calculated for the total effect (preventing smoking onset and initiating quit attempts).

### Ethics Approval and Consent to Participate

In accordance with Guidelines for Good Epidemiologic Practice [43], the study protocol was submitted for approval to the responsible ethics committee (Federal University of Ouro Preto, Brazil) and consent was obtained. All legal and data protection issues were discussed with the responsible authorities, and all participants were required to provide informed consent.

## Results

### Baseline Data

The mean age of the 2348 participants at baseline (Multimedia Appendix 1) was 14.8 years (range 12-21 years), and 50.72% (1191/2348) were female. At baseline, the survey identified 7.79% (183/2348) of participants as regular cigarette smokers, 12.05% (283/2348) as straw cigarette smokers, 4.64% (109/2348) as water pipe/hookah smokers, and 2.21% (52/2348) as e-cigarette smokers.

New derived variables were calculated for the analyses. In total, 14.78% (347/2348) of participants had smoked at least one regular or straw cigarette during the past 30 days, 5.88% (138/2348) had used a new tobacco product during the past 30 days (new tobacco product defined as water pipe/hookah or e-cigarettes), and 17.12% (402/2348) had used at least one of these during the past 30 days (regular/straw cigarettes, water pipe/hookah, or e-cigarette). The last characteristic was used as the criterion for the primary outcome and did not differ between the randomly assigned treatment groups at baseline (*P*=.83, Multimedia Appendix 1). However, there were strong influences of age, grade, and school performance on smoking behavior (Multimedia Appendix 2).

### Follow-Up Data

Data analyses were based on the originally assigned groups (Table 1). There were 744 students in the intervention group and 609 in the control group who participated in the survey at both baseline and at 12-month follow-up that could be identified (baseline sample N=2348; prospective sample: n=1353; lost to follow-up: n=995).

From baseline to 12-month follow-up, the smoking prevalence increased from 11.0% (67/609) to 20.9% (127/609) in the control group and from 14.1% (105/744) to 15.6% (116/744) in the intervention group (NNT=19), with an effect for female gender (control 12.4%, 43/346 to 18.8%, 65/346 vs intervention 13.1%, 54/411 to 14.6%, 60/411) and an even greater effect for males (control 9.1%, 24/263 to 23.6%, 62/263 vs intervention 15.3%, 51/333 to 16.8%, 56/333) (Table 1).

### Primary Outcome

The difference in the change in smoking prevalence between the control and intervention groups was statistically significant at 8.1% (95% CI 3.5%-12.7%, *P*<.001, *t* test calculated from estimated parameters). Smoking prevalence increased over the 12 months in both groups. However, in the intervention group, smoking only increased by 1.5% (95% CI −1.5% to 4.5%) compared to a 9.6% (95% CI 6.1%-13.1%) increase in the control group (Table 2).

Table 2 was calculated using the GENLINMIXED procedure (SPSS version 23) for a binomially distributed dependent variable with logit-link and an unstructured covariance matrix. Degrees of freedom were adjusted using the Satterthwaite approximation, and robust estimates were used. The class affiliation was used as random factor, meaning the clustered structure of the experimental design was taken into account. Age and academic performance at baseline were used for model adjustment. Academic performance was dichotomized as 0=good and very good; 1=reasonable, poor, and very poor. The (significant) influence of age and academic performance remained constant for the estimation of prevalence.

### Secondary Outcomes

The intervention effect could be explained by the EAT program preventing adolescents from starting smoking as well as encouraging quitting attempts, as can be seen in Table 3.

If the intervention and control group were additionally differentiated according to gender, there were four groups (Table 4). Smoking prevalence increased in all four groups. In the intervention group (both males and females), smoking increased by 1.5%. However, in the control group, the increase in smoking prevalence differed for males and females: 13.6% for males and 6.4% for females.

**Figure 2:**
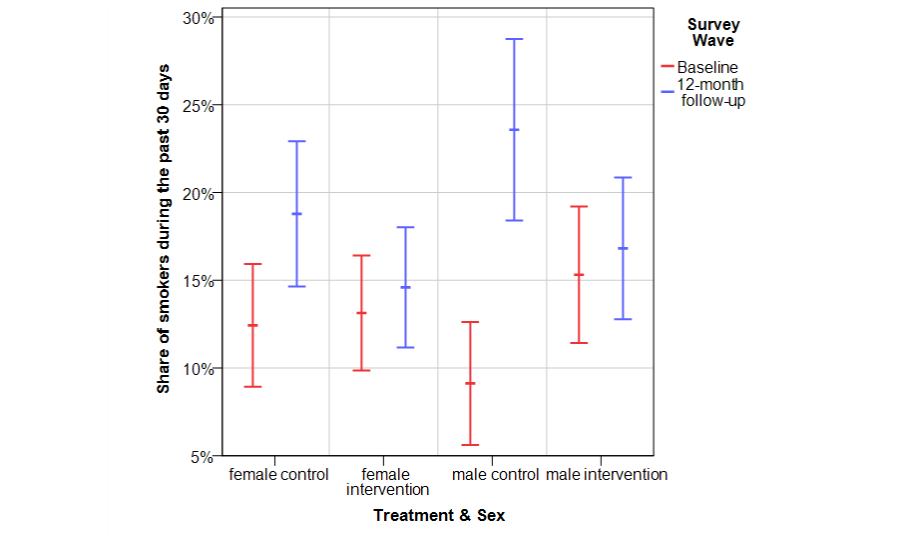
Effects of the intervention on smoking status by gender.

The difference in the change in smoking prevalence between the control and intervention groups was statistically significant among males (difference 12.1%, 95% CI 5.0%-19.1%, *P*<.001; *t* test calculated from estimated parameters). However, the difference in the change in smoking prevalence between the control and intervention groups was not statistically significant for females (difference 4.9%, 95% CI −0.7% to 10.4%, *P*=.09; *t* test calculated from estimated parameters). If only the control group was considered, the difference in the increase in smoking prevalence between males and females was significant (*P*=.049, *t* test). Figure 2 illustrates the gender-specific findings.

**Table 1:** Smoking prevalence at baseline (t1) and at the 6- (t2) and 12-month (t3) follow-ups.

Time point^a^				Total sample, n (%)		Intervention group, n (%)		Control group, n (%)		Number needed to treat
**Total**										
	t1			172/1353 (12.7)		105/744 (14.1)		67/609 (11.0)		
	t2			150/899 (16.7)		64/472 (13.6)		86/427 (20.1)		
	t3			243/1353 (18.0)		116/744 (15.6)		127/609 (20.9)		19/12^b^
**Gender**										
	**Female**									
		t1	97/757 (12.8)		54/411 (13.1)		43/346 (12.4)			
		t2	86/518 (16.6)		39/266 (14.7)		47/252 (18.7)			
		t3	125/757 (16.5)		60/411 (14.6)		65/346 (18.8)		24	
	**Male**									
		t1	75/596 (12.6)		51/333 (15.3)		24/263 (9.1)			
		t2	64/381 (16.8)		25/206 (12.1)		39/175 (22.3)			
		t3	118/596 (19.8)		56/333 (16.8)		62/263 (23.6)		15	
**Grade at baseline**										
	**7**									
		t1	25/341 (7.3)		19/213 (8.9)		6/128 (4.7)			
		t2	30/208 (14.4)		12/133 (9.0)		18/75 (24.0)			
		t3	56/341 (16.4)		28/213 (13.1)		28/128 (21.9)		11	
	**8**									
		t1	40/296 (13.5)		34/192 (17.7)		6/104 (5.8)			
		t2	38/185 (20.5)		24/114 (21.1)		14/71 (19.7)			
		t3	52/296 (17.6)		27/192 (14.1)		25/104 (24.0)		10	
	**9**									
		t1	14/86 (16.3)		6/34 (17.6)		8/52 (15.4)			
		t2	14/56 (25.0)		3/10 (30.0)		11/46 (23.9)			
		t3	19/86 (22.1)		9/34 (26.5)		10/52 (19.2)		−14	
	**10**									
		t1	48/317 (15.1)		18/116 (15.5)		30/201 (14.9)			
		t2	45/245 (18.4)		14/96 (14.6)		31/149 (20.8)			
		t3	61/317 (19.2)		23/116 (19.8)		38/201 (18.9)		−108	
	**11**									
		t1	45/313 (14.4)		28/189 (14.8)		17/124 (13.7)			
		t2	23/205 (11.2)		11/119 (9.2)		12/86 (14.0)			
		t3	55/313 (17.6)		29/189 (15.3)		26/124 (21.0)		18	
**Academic performance^c^** **(at baseline)**										
	**Very good**									
		t1	28/335 (8.4)		21/197 (10.7)		7/138 (5.1)			
		t2	24/216 (11.1)		12/122 (9.8)		12/94 (12.8)			
		t3	43/335 (12.8)		25/197 (12.7)		18/138 (13.0)		283	
	**Good**									
		t1	68/577 (11.8)		40/299 (13.4)		28/278 (10.1)			
		t2	54/411 (13.1)		23/212 (10.8)		31/199 (15.6)			
		t3	87/577 (15.1)		37/299 (12.4)		50/278 (18.0)		18	
	**Reasonable**									
		t1	64/363 (17.6)		34/201 (16.9)		30/162 (18.5)			
		t2	53/214 (24.8)		19/105 (18.1)		34/109 (31.2)			
		t3	91/363 (25.1)		42/201 (20.9)		49/162 (30.2)		11	
	**Poor**									
		t1	9/57 (15.8)		7/34 (20.6)		2/23 (8.7)			
		t2	14/43 (32.6)		8/25 (32.0)		6/18 (33.3)			
		t3	16/57 (28.1)		9/34 (26.5)		7/23 (30.4)		25	
	**Very poor**									
		t1	3/21 (14.3)		3/13 (23.1)		0/8 (0.0)			
		t2	5/15 (33.3)		2/8 (25.0)		3/7 (42.9)			
		t3	6/21 (28.6)		3/13 (23.1)		3/8 (37.5)		7	

^a^The case number at t1 and t3 corresponds to all participants at t3 (N=1353, the basis for the analysis of the primary outcome). For better comparability, only cases are used for t2 that were also present at t3 (N=899).

^b^The NNT is 12 if the baseline differences are taken into account.

^c^Academic performance was assessed by the students themselves through one single 5-point Likert scale item in the study questionnaire.

**Table 2:** Change in smoking prevalence in the intervention and control groups from baseline to 12-month follow-up.

Pairwise contrasts	Survey wave pairwise contrasts	Contrast estimate	SE	*t* (*df*)	*P* value^a^	95% CI^b^
Intervention	12-month follow-up from baseline	0.015	0.015	0.975 (883)	.33	−0.015, 0.045
Control	12-month follow-up from baseline	0.096	0.018	5.413 (714)	<.001	0.061, 0.131
Difference in change	Control and intervention groups	0.081	0.023	3.456 (1595)	<.001	0.035, 0.127

^a^The sequential Sidak adjusted significance level is .05.

^b^Confidence interval bounds are approximate.

**Table 3:** Nominal and percentage effects of the intervention on the smoking status (secondary outcomes) from baseline to 12-month follow-up (N=1353; *P*=.001 [Fisher test]).

Sample	Prospective smoking status (t1-t3)			
	Remained a nonsmoker (n=1041)	Started smoking (n=140)	Quit smoking (n=69)	Remained a smoker (n=103)
Control group, n (%)	460 (75.5)	82 (13.5)	22 (3.6)	45 (7.4)
Intervention group, n (%)	581 (78.1)	58 (7.8)	47 (6.3)	58 (7.8)

The change in smoking behavior was also examined separately for the intervention and control groups. In the control group, there was a 13.5% change from nonsmokers to smokers, but only a 3.6% change from smokers to nonsmokers. The proportion of smokers in the control group increased from 11.0% to 20.9%. This difference was statistically significant (*P*<.001, McNemar test). However, in the intervention group, the changes from smokers to nonsmokers and from nonsmokers to smokers were roughly balanced. The proportion of smokers increased moderately from 14.1% (105/744) to 15.6% (116/744), but the difference was not significant (*P*=.34, McNemar test). This suggests the intervention prevented a further increase in the proportion of smokers.

The differences in change in smoking prevalence from baseline to 12-month follow-up between the intervention and control groups were increased in participants with low school performance (Table 5). Dichotomized academic performance at baseline was used for the calculation (0=good or very good; 1= reasonable, poor, or very poor).

**Table 4:** Nominal and percentage effects of the intervention on smoking status by gender.

Sample			Prospective smoking status (t1-t3)						
			Remained a nonsmoker (n=1041)		Started smoking (n=140)		Quit smoking (n=69)		Remained a smoker (n=103)
**Control group, n (%)**									
	Total	460 (75.5)		82 (13.5)		22 (3.6)		45 (7.4)	
	Female	267 (77.2)		36 (10.4)		14 (4.0)		29 (8.4)	
	Male	193 (73.4)		46 (17.5)		8 (3.0)		16 (6.1)	
**Intervention group, n (%)**									
	Total	581 (78.1)		58 (7.8)		47 (6.3)		58 (7.8)	
	Female	324 (78.8)		33 (8.0)		27 (6.6)		27 (6.6)	
	Male	257 (77.2)		25 (7.5)		20 (6.0)		31 (9.3)	
**Total (n)**									
	Female	591		69		41		56	
	Male	450		71		28		47	

**Table 5:** Effect of school performance on the change in the prevalence of smoking, calculated with GENLINMIXED.

Academic performance and treatment		Survey wave pairwise contrasts	Contrast estimate	SE	*t* (*df*)	Adjusted significance^a^	95% CI^b^
**Not good**							
	Intervention	12-month follow-up from baseline	0.041	0.038	1.094 (356)	.28	−0.033, 0.115
	Control	12-month follow-up from baseline	0.138	0.033	4.141 (1155)	<.001	0.073, 0.203
**Good**							
	Intervention	12-month follow-up from baseline	0.002	0.014	0.145 (2077)	.89	−0.026, 0.031
	Control	12-month follow-up from baseline	0.077	0.020	3.936 (754)	<.001	0.039, 0.116

^a^The sequential Sidak adjusted significance level is .05.

^b^Confidence interval bounds are approximate.

## Discussion

### Principal Findings

To our knowledge, this is the first randomized trial on school-based tobacco prevention in Brazil that showed significant (*P*<.01) results in favor of the intervention. From baseline to the 12-month follow-up, the smoking prevalence increased from 11.0% to 20.9% in the control group and from 14.1% to 15.6% in the intervention group. This effect was increased for students with low educational background (ie, with low academic performance), which suggests that the intervention may contribute to reducing social inequalities among Brazilian adolescents, which are enhanced by tobacco addiction [44-48]. In addition, this study represents the first time the current (2015) EAT school curriculum was prospectively evaluated globally. Therefore, our findings have high relevance for the global EAT network (educationtobacco.org). In Brazil, EAT is currently established at 15 Brazilian medical schools, with more than 200 medical students volunteering to educate more than 8000 adolescents per year. Therefore, our findings also have high local relevance. Considering the NNT we found in this study (NNT=12 with baseline differences taken into account) and the current structure of EAT in Brazil (8000 adolescents covered annually), we can extrapolate that 667 Brazilian adolescents per year (8000/12) either quit smoking or do not start smoking due to EAT.

Local and international observations indicate that school-based preventions strongly motivated medical students within EAT for further tobacco control activities, such as engaging with politicians or improving medical education in regard to smoking cessation by organizing elective courses for their peers [13]. In Brazil, a national tobacco control award was organized by local medical students involved with EAT to recognize the best idea for enhancing tobacco control [49].

### Limitations

#### Lack of Biochemical Validation

Originally, we planned to biochemically validate our findings via carbon monoxide measurements with a portable CO analyzer “Smokerlyzer piCO+” (Bedfont Scientific, Maidstone, United Kingdom) that was purchased for the study [42]. However, random measurements were not possible as some schools and teachers refused to participate. This made systematic collection impossible, and we were not able to collect a sample for analysis.

#### Generalizability

As our research was not conducted multinationally, we cannot generalize our results to different countries and cultural backgrounds. However, the similarity between the results found here and the ones found in our German studies [17-19] increases the international validity of our research. Also, as this study was performed in the setting of Brazilian public schools, the results might not be generalizable to private schools. The fact that there were some students aged 18 to 21 years in our study (the normal expected age range for grades 7-11 would be 12-17 years) may reflect higher rates of grade retention in public schools. Nevertheless, after excluding those participants aged 18 to 21 years (117/2348), we repeated all statistical analyses (data not shown) and noted that the significant results found for the total number of students remained the same.

### Comparison With Prior Work in Brazil

This is the first randomized trial on school-based tobacco prevention in Brazil that showed significant (*P*<.01) results in favor of the intervention. Prior work failed to show such an effect mainly due to lack of sample size, funding, and incompletely implemented interventions [11,12]. By using motivated volunteer medical students, we were not dependent on the participation and training of teachers but had a preselected enthusiastic team that achieved complete implementation in the intervention classes. However, although the use of medical students has many benefits [13], it limits the number of adolescents that may be reached by the intervention.

### Conclusions

The EAT intervention prevents smoking by encouraging quitting and preventing smoking onset, especially among males and students with low educational background.

## Appendix

Multimedia Appendix 1 Descriptive characteristics at baseline: all cases including dropouts.

Multimedia Appendix 2 Some bivariate relationships at baseline: all cases including dropouts.

Multimedia Appendix 3 CONSORT‐EHEALTH checklist (V 1.6.1).

## Abbreviations

EAT: Education Against Tobacco
NNT: number needed to treat
OR: odds ratio

## References

[1] Pinto M, Bardach A, Palacios A, Biz A, Alcaraz A, Rodríguez B, Augustovski F, Pichon-Riviere A (2017). Carga de doença atribuível ao uso do tabaco no Brasil e potencial impacto do aumento de preços por meio de impostos. Documento técnico IECS N° 21.

[2] Barreto S, Giatti L, Oliveira-Campos M, Andreazzi M, Malta DC (2014). Experimentation and use of cigarette and other tobacco products among adolescents in the Brazilian state capitals (PeNSE 2012). Rev Bras Epidemiol.

[3] Thomas R, McLellan J, Perera R (2015). Effectiveness of school-based smoking prevention curricula: systematic review and meta-analysis. BMJ Open.

[4] Peirson L, Ali M, Kenny M, Raina P, Sherifali D (2016). Interventions for prevention and treatment of tobacco smoking in school-aged children and adolescents: a systematic review and meta-analysis. Prev Med.

[5] Hefler M, Liberato S, Thomas DP (2017). Incentives for preventing smoking in children and adolescents. Cochrane Database Syst Rev.

[6] Bast L, Due P, Ersbøll AK, Damsgaard M, Andersen A (2017). Association of school characteristics and implementation in the X:IT Study-a school-randomized smoking prevention program. J Sch Health.

[7] Midford R, Cahill H, Lester L, Foxcroft D, Ramsden R, Venning L (2016). Smoking prevention for students: findings from a three-year program of integrated harm minimization school drug education. Subst Use Misuse.

[8] Garnham-Lee K, Trigwell J, McGee CE, Knowles Z, Foweather L (2016). Impact and acceptability of the coach and teacher training within a school-based sport-for-health smoking prevention intervention: SmokeFree Sports. J Child Adoles Subst.

[9] Brown N, Luckett T, Davidson P, DiGiacomo M (2017). Family-focussed interventions to reduce harm from smoking in primary school-aged children: a systematic review of evaluative studies. Prev Med.

[10] Chan S, Cheung Y, Fong D, Emmons K, Leung A, Leung D, Lam TH (2017). Family-based smoking cessation intervention for smoking fathers and nonsmoking mothers with a child: a randomized controlled trial. J Pediatr.

[11] do Nascimento MO, De Micheli D (2015). Evaluation of different school-based preventive interventions for reducing the use of psychotropic substances among students: a randomized study. Cien Saude Colet.

[12] Malcon M, Menezes A, Assunção M, Neutzling M, Challal P (2011). Efetividade de uma intervenção educacional em tabagismo entre adolescentes escolares. Rev Bras Epidemiol.

[13] Brinker T, Buslaff F, Haney C, Gaim B, Haney A, Schmidt S, Silchmüller MP, Taha L, Jakob L, Baumert HM, Hallmann M, Heckl M, Alfitian J, Brieske CM, Divizieva EP, Wilhelm J, Hillebrand G, Penka D, Raveendranathan S, Suhre JL, Netzwerk Aufklärung gegen Tabak (2018). [The global medical network Education Against Tobacco-voluntary tobacco prevention made in Germany]. Bundesgesundheitsblatt Gesundheitsforschung Gesundheitsschutz.

[14] Brinker TJ, Seeger W (2015). Photoaging mobile apps: a novel opportunity for smoking cessation?. J Med Internet Res.

[15] Education Against Tobacco.

[16] Brinker T, Seeger W, Buslaff F (2016). Photoaging mobile apps in school-based tobacco prevention: the mirroring approach. J Med Internet Res.

[17] Brinker T, Stamm-Balderjahn S, Seeger W, Klingelhöfer D, Groneberg DA (2015). Education Against Tobacco (EAT): a quasi-experimental prospective evaluation of a multinational medical-student-delivered smoking prevention programme for secondary schools in Germany. BMJ Open.

[18] Brinker T, Stamm-Balderjahn S, Seeger W, Groneberg DA (2014). Education Against Tobacco (EAT): a quasi-experimental prospective evaluation of a programme for preventing smoking in secondary schools delivered by medical students: a study protocol. BMJ Open.

[19] Brinker T, Owczarek A, Seeger W, Groneberg D, Brieske C, Jansen P, Klode J, Stoffels I, Schadendorf D, Izar B, Fries FN, Hofmann FJ (2017). A medical student-delivered smoking prevention program, Education Against Tobacco, for secondary schools in Germany: randomized controlled trial. J Med Internet Res.

[20] Bernstein S, Yu S, Post L, Dziura J, Rigotti NA (2013). Undertreatment of tobacco use relative to other chronic conditions. Am J Public Health.

[21] Raupach T, Falk J, Vangeli E, Schiekirka S, Rustler C, Grassi M, Pipe A, West R (2014). Structured smoking cessation training for health professionals on cardiology wards: a prospective study. Eur J Prev Cardiol.

[22] Anders S, Strobel L, Krampe H, Raupach T (2013). [Do final-year medical students know enough about the treatment of alcohol use disorders and smoking?]. Dtsch Med Wochenschr.

[23] Flett K, Grogan S, Clark-Carter D, Gough B, Conner M (2017). Male smokers' experiences of an appearance-focused facial-ageing intervention. J Health Psychol.

[24] Jiwa M, Burford O, Parsons R (2015). Preliminary findings of how visual demonstrations of changes to physical appearance may enhance weight loss attempts. Eur J Public Health.

[25] Mahler H, Kulik J, Butler H, Gerrard M, Gibbons FX (2008). Social norms information enhances the efficacy of an appearance-based sun protection intervention. Soc Sci Med.

[26] Olson A, Gaffney C, Starr P, Dietrich AJ (2008). The impact of an appearance-based educational intervention on adolescent intention to use sunscreen. Health Educ Res.

[27] Owen A, Grogan S, Clark-Carter D (2016). Effects of an appearance-focussed versus a health-focussed intervention on men's attitudes towards UV exposure. Int J Men Health.

[28] Stapleton J, Turrisi R, Hillhouse J, Robinson J, Abar B (2010). A comparison of the efficacy of an appearance-focused skin cancer intervention within indoor tanner subgroups identified by latent profile analysis. J Behav Med.

[29] Tuong W, Armstrong AW (2014). Effect of appearance-based education compared with health-based education on sunscreen use and knowledge: a randomized controlled trial. J Am Acad Dermatol.

[30] Brinker T, Holzapfel J, Baudson T, Sies K, Jakob L, Baumert H, Heckl M, Cirac A, Suhre J, Mathes V, Fries FN, Spielmann H, Rigotti N, Seeger W, Herth F, Groneberg DA, Raupach T, Gall H, Bauer C, Marek P, Batra A, Harrison CH, Taha L, Owczarek A, Hofmann FJ, Thomas R, Mons U, Kreuter M (2016). Photoaging smartphone app promoting poster campaign to reduce smoking prevalence in secondary schools: the Smokerface Randomized Trial: design and baseline characteristics. BMJ Open.

[31] Brinker T, Schadendorf D, Klode J, Cosgarea I, Rösch A, Jansen P, Stoffels I, Izar B (2017). Photoaging mobile apps as a novel opportunity for melanoma prevention: pilot study. JMIR Mhealth Uhealth.

[32] Burford O, Smith M, Jiwa M, Carter O (2008). Photoageing Intervention (PAINT): a proposal for a randomised controlled trial in Australian primary care. AMJ.

[33] Eastabrook S, Chang P, Taylor MF (2018). Melanoma risk: adolescent females' perspectives on skin protection pre/post-viewing a ultraviolet photoaged photograph of their own facial sun damage. Glob Health Promot.

[34] Faria B, Brieske C, Cosgarea I, Omlor A, Fries F, de Faria CO, Lino H, Oliveira A, Lisboa O, Klode J, Schadendorf D, Bernardes-Souza B, Brinker TJ (2017). A smoking prevention photoageing intervention for secondary schools in Brazil delivered by medical students: protocol for a randomised trial. BMJ Open.

[35] Mahler H, Kulik J, Gerrard M, Gibbons FX (2013). Effects of photoaging information and UV photo on sun protection intentions and behaviours: a cross-regional comparison. Psychol Health.

[36] Lo Presti L, Chang P, Taylor MF (2014). Young Australian adults' reactions to viewing personalised UV photoaged photographs. Australas Med J.

[37] Brinker T, Heckl M, Gatzka M, Heppt M, Resende Rodrigues H, Schneider S, Sondermann W, de Almeida E Silva C, Kirchberger M, Klode J, Enk AH, Knispel S, von Kalle C, Stoffels I, Schadendorf D, Nakamura Y, Esser S, Assis A, Bernardes-Souza B (2018). A skin cancer prevention facial-aging mobile app for secondary schools in Brazil: appearance-focused interventional study. JMIR Mhealth Uhealth.

[38] Brinker T, Brieske C, Esser S, Klode J, Mons U, Batra A, Rüther T, Seeger W, Enk A, von Kalle C, Berking C, Heppt MV, Gatzka MV, Bernardes-Souza B, Schlenk RF, Schadendorf D (2018). A face-aging app for smoking cessation in a waiting room setting: pilot study in an HIV outpatient clinic. J Med Internet Res.

[39] Brinker T, Klode J, Esser S, Schadendorf D (2018). Facial-aging app availability in waiting rooms as a potential opportunity for skin cancer prevention. JAMA Dermatol.

[40] Baudson T, Weber K, Freund PA (2016). More than only skin deep: appearance self-concept predicts most of secondary school students' self-esteem. Front Psychol.

[41] Burford O, Jiwa M, Carter O, Parsons R, Hendrie D (2013). Internet-based photoaging within Australian pharmacies to promote smoking cessation: randomized controlled trial. J Med Internet Res.

[42] Xavier L, Bernardes-Souza B, Lisboa O, Seeger W, Groneberg D, Tran T, Fries F, Corrêa PC, Brinker TJ (2017). A medical student-delivered smoking prevention program, Education Against Tobacco, for secondary schools in Brazil: study protocol for a randomized trial. JMIR Res Protoc.

[43] BMBF Gesundheitsforschung (2004). Deutsche Arbeitsgemeinschaft fuer Epidemiologie.

[44] Kuntz B, Waldhauer J, Moor I, Rathmann K, Richter M, Orth B, Piontek D, Kraus L, Zeiher J, Lampert T (2018). [Trends in educational inequalities in smoking among adolescents in Germany: evidence from four population-based studies]. Bundesgesundheitsblatt Gesundheitsforschung Gesundheitsschutz.

[45] Hoebel J, Kuntz B, Kroll L, Finger J, Zeiher J, Lange C, Lampert T (2018). Trends in absolute and relative educational inequalities in adult smoking since the early 2000s: the case of Germany. Nicotine Tob Res.

[46] Kuntz B, Zeiher J, Hoebel J, Lampert T (2016). Soziale Ungleichheit, Rauchen und Gesundheit. Suchttherapie.

[47] Kuntz B, Lampert T (2016). Smoking and passive smoke exposure among adolescents in Germany. Dtsch Arztebl Int.

[48] Kuntz B, Hoebel J, Lampert T (2014). [Educational differences in smoking and smoking cessation among young adults. Results from the "German Health Update" (GEDA) Study 2009 and 2010]. Gesundheitswesen.

[49] Corrêa PCRP, Pereira RMOS, Temporao JG, Cavalcante TM, Lisboa OC, Azevedo LG, Brinker TJ, Bernardes-Souza B (2019). EAT-Brazil Award for Tobacco Control: a brief description of the profile of the proposals submitted in the 1st edition of the award. Revista da Associação Médica Brasileira.

