# Supplementary material for: A Smoking Prevention Program Delivered by Medical Students to Secondary Schools in Brazil Called “Education Against Tobacco”: Randomized Controlled Trial
Source: J Med Internet Res. 2019 Feb 21;21(2):e12854. doi: 10.2196/12854 (PMC6416894; doi:10.2196/12854)
Supplement: Multimedia Appendix 1 [file jmir_v21i2e12854_app1.pdf]

Multimedia Appendix 1. Descriptive characteristics at baseline: all cases including dropouts

Variable	Entire sample n/N (%)	Intervention group n/N (%)	Control group n/N (%)	p-value
**Number of students**	2348/2348 (100)	1267/2348 (54)	1081/2348 (46)	0.000[1]
**Number of classes**	110/110 (100)	59/110 (53.6)	51/110 (46.4)	0.446[1]
**Gender**	2348/2348 (100)			
Female	1191/2348 (50.7)	630/1267 (49.7)	561/1081 (51.9)	0.301[2]
Male	1157/2348 (49.3)	637/1267(50.3)	520/1081 (48.1)	
**Age, years**	2348/2348 (100)	1267/2348 (54)	1081/2348 (46)	
	M=14.8 SD=1.86	M=14.7 SD=1.88	M=14.9 SD=1.84	0.005[3]
**Grade**	2348/2348 (100)	1267/2348 (54)	1081/2348 (46)	
7	582/2348 (24.8)	327/1267 (25.8)	255/1081 (23.6)	
8	485/2348 (20.7)	319/1267 (25.2)	166/1081 (15.4)	
9	150/2348 (6.4)	57/1267 (4.5)	93/1081 (8.6)	0.000[4]
10	618/2348 (26.3)	258/1267 (20.4)	360/1081 (33.3)	
11	513/2348 (21.8)	306/1267 (24.2)	207/1081 (19.1)	
**Academic**				

**performance**				
Very good	520/2348 (22.1)	297/1267 (23.4)	223/1081 (20.6)	0.479[4]
Good	946/2348 (40.3)	493/1267 (38.9)	453/1081 (41.9)	
Reasonable	677/2348 (28.8)	368/1267 (29.0)	309/1081 (28.6)	
Poor	129/2348 (5.5)	69/1267 (5.4)	60/1081 (5.6)	
Very poor	76/2348 (3.2)	40/1267 (3.2)	36/1081 (3.3)	
**Which tobacco products have you used at least once in the past 30 days? (current use)**				
Regular cigarettes	183/2348 (7.8)	100/1267 (7.9)	83/1081 (7.7)	0.877[2]
Straw cigarettes	284/2348 (12.1)	150/1267 (11.8)	134/1081 (12.4)	0.703[2]
Water pipe/hookah	109/2348 (4.6)	66/1267 (5.2)	43/1081 (4.0)	0.169[2]
E-cigarette	52/2348 (2.2)	34/1267 (2.7)	18/1081 (1.7)	0.121[2]
Marijuana	235/2348 (10.0)	127/1267 (10.0)	108/1081 (10.0)	1.000[2]
**At least one regular or straw cigarette during the past 30 days**	347/2348 (14.8)	183/1267 (14.4)	164/1081 (15.2)	0.641[2]
**New tobacco products during the past 30 days**	138/2348 (5.9)	88/1267 (6.9)	50/1081 (4.6)	0.018[2]
Objective criterion: At least one	402/2348 (17.1)	219/1267 (17.3)	183/1081 (16.9)	0.826[2]

product during the past 30 days (regular/straw cigarettes, water pipe or e-cigarette)				
Female	194/1191 (16.3)	102/630 (16.2)	92/561 (16.4)	0.937[2]
Male	208/1157 (18.0)	117/637 (18.4)	91/520 (17.5)	0.758[2]

SD, standard deviation.

[1] Chi-square goodness-of-fit test

[2] Fisher's exact test

[3] T-test for independent groups

[4] Pearson chi-square test
