# Supplementary material for: A Smoking Prevention Program Delivered by Medical Students to Secondary Schools in Brazil Called “Education Against Tobacco”: Randomized Controlled Trial
Source: J Med Internet Res. 2019 Feb 21;21(2):e12854. doi: 10.2196/12854 (PMC6416894; doi:10.2196/12854)
Supplement: Multimedia Appendix 2 [file jmir_v21i2e12854_app2.pdf]

Multimedia Appendix 2. Some bivariate relationships at baseline: all cases including dropouts

		At least one regular or straw cigarette during the past 30 days	New tobacco products during the past 30 days	At least one product during the past 30 days
		Smokers' share	Smokers' share	Smokers' share
**Gender**	Total (N=2348)	14.8%	5.9%	17.1%
	Female (n=1191)	14.4%	4.4%	16.3%
	Male (n=1157)	15.1%	7.4%	18.0%
	Odds ratio	1.06	1.76	1.13
	Significance	p=0.641	p=0.002	p=0.278
**Age at base-line, years (grouped)**	12 (n=352)	3.7%	2.0%	4.5%
	13 (n=381)	8.9%	6.3%	11.5%
	14 (n=290)	15.9%	7.6%	19.0%
	15 (n=366)	18.3%	7.4%	21.9%
	16 (n=475)	16.2%	4.4%	18.1%
	17 (n=367)	22.1%	6.3%	24.0%
	18+ (n=117)	24.8%	12.0%	28.2%
	Odds ratio	1.31	1.12	1.28
	Significance	p=0.000	p=0.16	p=0.000
**Grade at baseline**	7 (n=582)	7.4%	5.0%	10.0%
	8 (n=485)	13.4%	7.2%	16.3%
	9 (n=150)	18.7%	9.3%	24.7%
	10 (n=618)	19.3%	5.3%	20.9%
	11 (n=513)	17.9%	5.3%	19.3%
	Odds ratio	1.26	0.98	1.19
	Significance	p=0.000	p=0.743	p=0.000
**Academic performance (at baseline)**	Very good (n=520)	8.3%	2.7%	9.8%
	Good (n=946)	11.7%	4.8%	13.7%
	Reasonable (n=677)	20.1%	8.4%	23.2%
	Poor (n=129)	27.9%	7.8%	30.2%
	Very poor (n=76)	27.6%	15.8%	32.9%
	Odds ratio	1.56	1.57	1.57
	Significance	p=0.000	p=0.000	p=0.000

Odds ratios and significance computed using binary logistic regression with smoking behavior as dependent dichotomous variables. All predictors were considered metric, and gender coded as 0 (female) and 1 (male). Separate models were calculated for each predictor and each smoking variable.
