# Supplementary material for: A Smoking Prevention Program Delivered by Medical Students to Secondary Schools in Brazil Called “Education Against Tobacco”: Randomized Controlled Trial
Source: J Med Internet Res. 2019 Feb 21;21(2):e12854. doi: 10.2196/12854 (PMC6416894; doi:10.2196/12854)
Supplement: Multimedia Appendix 3 [file jmir_v21i2e12854_app3.pdf]

CONSORT-EHEALTH Checklist V1.6.2 Report	Manuscript Number	12854
**(based on CONSORT-EHEALTH V1.6), available at [http://tinyurl.com/consort-ehealth-v1-6].**		
**Date completed**		
2/20/2019 14:13:00		
**by**		
Breno Bernardes-Souza		
A Smoking Prevention Program Delivered by Medical Students to Secondary Schools in Brazil Called “Education Against Tobacco”: Randomized Controlled Trial		
TITLE		
**1a-i) Identify the mode of delivery in the title**		
Yes: "A Smoking Prevention Program Delivered by Medical Students to Secondary Schools"		
**1a-ii) Non-web-based components or important co-interventions in title**		
**1a-iii) Primary condition or target group in the title**		
Yes: "A Smoking Prevention Program Delivered by Medical Students to Secondary Schools in Brazil"		
ABSTRACT		
**1b-i) Key features/functionalities/components of the intervention and comparator in the METHODS section of the ABSTRACT**		
Yes: "The study groups comprised randomized classes receiving the standardized EAT intervention (90 minutes of mentoring in a classroom setting) and control classes in the same schools (no intervention)."		
**1b-ii) Level of human involvement in the METHODS section of the ABSTRACT**		
**1b-iii) Open vs. closed, web-based (self-assessment) vs. face-to-face assessments in the METHODS section of the ABSTRACT**		
**1b-iv) RESULTS section in abstract must contain use data**		
**1b-v) CONCLUSIONS/DISCUSSION in abstract for negative trials**		
INTRODUCTION		
**2a-i) Problem and the type of system/solution**		
Yes. Besides addressing these points in our manuscript, we also cite our previously published study protocol (JMIR Research Protocols, 2017) which addresses all of these points in more detail. Quotes from the manuscript:<br><br>"Education Against Tobacco (EAT) is a network of volunteer medical students and physicians from more than 80 medical schools in 14 countries worldwide that was founded in Germany in 2012 [13]. The network has its roots in school-based interventions delivered by medical students. These interventions cost approximately US $20 per participating class and can reach up to 50,000 students per year worldwide."<br><br>"This study aimed to determine the long-term effectiveness of the school-based EAT intervention in reducing smoking prevalence among secondary school students in Brazil, as per the study protocol [42]."		
**2a-ii) Scientific background, rationale: What is known about the (type of) system**		
Yes. Besides addressing these points in our manuscript, we also cite our previously published study protocol (JMIR Research Protocols, 2017) which addresses all of these points in more detail. Quotes from the manuscript:<br><br>"Most school-based tobacco control programs are ineffective, but data from Brazil remain scarce [3-5]. Recent trials on tobacco prevention in the school setting have focused on including school teachers in the intervention [6-8], with others involving families [9,10]. However, these studies concluded that the students’ environment (ie, peer group as well as parental behavior and school policies) plays a role in smoking initiation in adolescence."<br><br>Regardint the EAT intervention: "A German quasi-experimental study showed the school-based intervention resulted in a significant reduction in smoking prevalence among secondary school students at 6-month follow-up [17,18]. A randomized follow-up study in Germany indicated effectiveness at the 12-month follow-up; however, the results were not significant because of a large loss-to-follow-up effect [19]."		
**Does your paper address CONSORT subitem 2b?**		
Yes: "This study aimed to determine the long-term effectiveness of the school-based EAT intervention in reducing smoking prevalence among secondary school students in Brazil, as per the study protocol [42]."		
METHODS		
**3a) CONSORT: Description of trial design (such as parallel, factorial) including allocation ratio**		
Yes. Besides addressing this point in our manuscript, we also cite our previously published study protocol (JMIR Research Protocols, 2017) which addresses it in more detail. Quote from the manuscript:<br><br>"A randomized controlled trial was conducted among 2384 adolescents in grades 7 to 11 from secondary schools in Brazil from February 2017 to June 2018 (Figure 1). All predefined time points were met. Details of the study design and the development of the questionnaire are outlined in our previously published study protocol [42]."<br><br>Figure 1 details the study design and flow of participants.		
**3b) CONSORT: Important changes to methods after trial commencement (such as eligibility criteria), with reasons**		
There were no important changes to methods after trial commencement. The potential limitations are discussed in the "Discussion" section.		
**3b-i) Bug fixes, Downtimes, Content Changes**		
**4a) CONSORT: Eligibility criteria for participants**		
Yes. Besides addressing it in our manuscript, we also cite our previously published study protocol (JMIR Research Protocols, 2017) which addresses the eligibility criteria in more detail. Quote from the manuscript:<br><br>"Students in grades 7 to 11 at secondary public schools were eligible to participate in this study."		
**4a-i) Computer / Internet literacy**		
**4a-ii) Open vs. closed, web-based vs. face-to-face assessments:**		
Yes. The participants (school adolescents) were recruited from public secondary schools in Brazil. The trial included face-to-face components (the entire intervention is described in detail in our previously published study protocol, besides being entirely available in our website). Pencil-and-paper questionnaires were used for data collection.		
**4a-iii) Information giving during recruitment**		
**4b) CONSORT: Settings and locations where the data were collected**		
A pencil-and-paper questionnaire was used for data collection. Class teachers supervised their classes during the completion of the questionnaire and sealed them right after completion for confidentiality reasons. The subsection "Data Collection" of our previously publised study protocol (JMIR Research Protocols, 2017) addresses it in more detail.		
**4b-i) Report if outcomes were (self-)assessed through online questionnaires**		
N/A. Only pencil-and-paper questionnaires were used.		
**4b-ii) Report how institutional affiliations are displayed**		
**5) CONSORT: Describe the interventions for each group with sufficient details to allow replication, including how and when they were actually administered**		
**5-i) Mention names, credential, affiliations of the developers, sponsors, and owners**		
**5-ii) Describe the history/development process**		
**5-iii) Revisions and updating**		
**5-iv) Quality assurance methods**		

5-v) Ensure replicability by publishing the source code, and/or providing screenshots/screen-capture video, and/or providing flowcharts of the algorithms used

5-vi) Digital preservation

5-vii) Access

N/A

5-viii) Mode of delivery, features/functionalities/components of the intervention and comparator, and the theoretical framework

Yes. This is a medical student-delivered smoking prevention intervention for school adolescents (secondary schools). All components and theoretical framework of the intervention are described in detail in our previously published study protocol, besides being entirely available in our website, so the intervention can be easily reproduced anywhere in the world.

5-ix) Describe use parameters

5-x) Clarify the level of human involvement

5-xi) Report any prompts/reminders used

N/A

5-xii) Describe any co-interventions (incl. training/support)

N/A

6a) CONSORT: Completely defined pre-specified primary and secondary outcome measures, including how and when they were assessed

Yes. Our primary and secondary outcomes, as well as the statistical analysis that would be done, were clearly pre-specified in our previously published study protocol (JMIR Research Protocols, 2017). We also made it clear in the current manuscript:

"The primary outcome was the difference in smoking prevalence from baseline (t1) to 12 months of follow-up (t3) in the control group versus the difference from t1 to t3 in the intervention group. The differences in smoking behavior (smoking onset, quit attempts) between the two groups were studied as secondary outcomes, along with gender-specific effects."

6a-i) Online questionnaires: describe if they were validated for online use and apply CHERRIES items to describe how the questionnaires were designed/deployed

6a-ii) Describe whether and how “use” (including intensity of use/dosage) was defined/measured/monitored

6a-iii) Describe whether, how, and when qualitative feedback from participants was obtained

6b) CONSORT: Any changes to trial outcomes after the trial commenced, with reasons

A pencil-and-paper questionnaire was used for data collection. Class teachers supervised their classes during the completion of the questionnaire and sealed them right after completion for confidentiality reasons. The subsection "Data Collection" of our previously publised study protocol (JMIR Research Protocols, 2017) addresses it in more detail.

7a) CONSORT: How sample size was determined

7a-i) Describe whether and how expected attrition was taken into account when calculating the sample size

7b) CONSORT: When applicable, explanation of any interim analyses and stopping guidelines

Yes. Our primary and secondary outcomes, as well as the statistical analysis that would be done, were clearly pre-specified in our previously published study protocol (JMIR Research Protocols, 2017). We also made it clear in the current manuscript:

"The primary outcome was the difference in smoking prevalence from baseline (t1) to 12 months of follow-up (t3) in the control group versus the difference from t1 to t3 in the intervention group. The differences in smoking behavior (smoking onset, quit attempts) between the two groups were studied as secondary outcomes, along with gender-specific effects."

8a) CONSORT: Method used to generate the random allocation sequence

Randomization was externally and centrally performed via computer by a statistician from the University of Gießen, Germany, on the class level within each school (group allocation was 1:1). The subsection "Randomization" of our previously published study protocol (JMIR Research Protocols, 2017) addresses it in detail.

8b) CONSORT: Type of randomisation; details of any restriction (such as blocking and block size)

Yes, as mentioned in the subitem 8a.

9) CONSORT: Mechanism used to implement the random allocation sequence (such as sequentially numbered containers), describing any steps taken to conceal the sequence until interventions were assigned

Yes, as mentioned in the subitem 8a.

10) CONSORT: Who generated the random allocation sequence, who enrolled participants, and who assigned participants to interventions

Yes, as mentioned in the subitem 8a.

11a) CONSORT: Blinding - If done, who was blinded after assignment to interventions (for example, participants, care providers, those assessing outcomes) and how

11a-i) Specify who was blinded, and who wasn’t

Due to its design (educational intervention vs no intervention), the study was not blinded.

11a-ii) Discuss e.g., whether participants knew which intervention was the “intervention of interest” and which one was the “comparator”

11b) CONSORT: If relevant, description of the similarity of interventions

N/A

12a) CONSORT: Statistical methods used to compare groups for primary and secondary outcomes

Yes. Besides addressing the statistical methods in our manuscript, we also cite our previously published study protocol (JMIR Research Protocols, 2017) which had already defined the statistical analysis that would be undertaken. Quote from the manuscript:

"We used chi-square tests, t tests, and Fisher exact tests to examine baseline differences. The effects of predictors (gender and social characteristics) on smoking behavior at 12 months were calculated using robust panel logistic regression analysis. The significance level was set at 5% for t tests (two-sided) with 95% CIs (two-sided). Statistical analyses were performed using SPSS version 23 (IBM Corp, Armonk, NY, USA). The group allocation of the study sample was based on class level. Statistically robust panel logistic regression was used (SPSS GENLINMIXED procedure) to account for clustering. This procedure was also used to calculate the difference in the smoking prevalence from baseline to 12-month follow-up in the control group versus that in the intervention group (primary outcome). The number needed to treat (NNT) was calculated for the total effect (preventing smoking onset and initiating quit attempts)."

12a-i) Imputation techniques to deal with attrition / missing values

All the attrition analysis are described in detail in the "Attrition Analysis" subsection of the manuscript.

12b) CONSORT: Methods for additional analyses, such as subgroup analyses and adjusted analyses

Yes, as mentioned above in the subitem 12a.

RESULTS

13a) CONSORT: For each group, the numbers of participants who were randomly assigned, received intended treatment, and were analysed for the primary outcome

Yes, the "Results" section of our manuscript addresses all of it in detail. For example:

"Data analyses were based on the originally assigned groups (Table 1). There were 744 students in the intervention group and 609 in the control group who participated in the survey at both baseline and at 12-month follow-up that could be identified (baseline sample N=2348; prospective sample: n=1353; lost to follow-up: n=995)."

13b) CONSORT: For each group, losses and exclusions after randomisation, together with reasons

Yes. We addressed the losses after randomisation as well as we used statistical analysis to evaluate attrition bias. We also made it clear that "Reasons for loss to follow-up included identifier code not assignable, change of school, unauthorized absence from class (truancy), illness, or grade retention."

13b-i) Attrition diagram

14a) CONSORT: Dates defining the periods of recruitment and follow-up

Yes: "Baseline data (t1) were collected from February 2017 to May 2017. Follow-up data (t2 and t3) were collected 6 and 12 months after that (from August 2017 to June 2018)."

14a-i) Indicate if critical “secular events” fell into the study period

14b) CONSORT: Why the trial ended or was stopped (early)

N/A		
**15) CONSORT: A table showing baseline demographic and clinical characteristics for each group**		
Yes: this data is in Table 1, as well as in Multimedia Appendix 1 and Multimedia Appendix 2.		
**15-i) Report demographics associated with digital divide issues**		
N/A		
**16a) CONSORT: For each group, number of participants (denominator) included in each analysis and whether the analysis was by original assigned groups**		
**16-i) Report multiple “denominators” and provide definitions**		
Yes. The denominator is present throughout all tables and text of the Results section.		
**16-ii) Primary analysis should be intent-to-treat**		
**17a) CONSORT: For each primary and secondary outcome, results for each group, and the estimated effect size and its precision (such as 95% confidence interval)**		
Yes. The 95% CI was calculated and is present throughout the tables and text of the Results section.		
**17a-i) Presentation of process outcomes such as metrics of use and intensity of use**		
**17b) CONSORT: For binary outcomes, presentation of both absolute and relative effect sizes is recommended**		
Yes: it was calculated and presented accordingly.		
**18) CONSORT: Results of any other analyses performed, including subgroup analyses and adjusted analyses, distinguishing pre-specified from exploratory**		
Yes. We address all these results in the "Results" section: all analyses were pre-specified and are detailed both in this manuscript as well as in our previously published study protocol (JMIR Research Protocols, 2017).		
**18-i) Subgroup analysis of comparing only users**		
**19) CONSORT: All important harms or unintended effects in each group**		
No harms/unintended effects were identified.		
**19-i) Include privacy breaches, technical problems**		
**19-ii) Include qualitative feedback from participants or observations from staff/researchers**		
DISCUSSION		
**20) CONSORT: Trial limitations, addressing sources of potential bias, imprecision, multiplicity of analyses**		
**20-i) Typical limitations in ehealth trials**		
Yes: our manuscript has a "Limitations" subsection that addresses it, for example:<br><br>"As our research was not conducted multinationally, we cannot generalize our results to different countries and cultural backgrounds. However, the similarity between the results found here and the ones found in our German studies [17-19] increases the international validity of our research. Also, as this study was performed in the setting of Brazilian public schools, the results might not be generalizable to private schools."		
**21) CONSORT: Generalisability (external validity, applicability) of the trial findings**		
**21-i) Generalizability to other populations**		
**21-ii) Discuss if there were elements in the RCT that would be different in a routine application setting**		
**22) CONSORT: Interpretation consistent with results, balancing benefits and harms, and considering other relevant evidence**		
**22-i) Restate study questions and summarize the answers suggested by the data, starting with primary outcomes and process outcomes (use)**		
Yes: "To our knowledge, this is the first randomized trial on school-based tobacco prevention in Brazil that showed significant ($P<.01$) results in favor of the intervention. From baseline to the 12-month follow-up, the smoking prevalence increased from 11.0% to 20.9% in the control group and from 14.1% to 15.6% in the intervention group. This effect was increased for students with low educational background (ie, with low academic performance), which suggests that the intervention may contribute to reducing social inequalities among Brazilian adolescents, which are enhanced by tobacco addiction [44-48]."		
**22-ii) Highlight unanswered new questions, suggest future research**		
Other information		
**23) CONSORT: Registration number and name of trial registry**		
Yes. Trial Registration: ClinicalTrials.gov NCT02725021		
**24) CONSORT: Where the full trial protocol can be accessed, if available**		
Yes. The full trial protocol is cited in our manuscript and it was publised at JMIR Research Protocols in 2017: doi:10.2196/resprot.7134		
**25) CONSORT: Sources of funding and other support (such as supply of drugs), role of funders**		
A grant from the German Heart Foundation provided funding for the Smokerface App and a grant from the German Center for Lung Research funded the CO measurement device and the Samsung tablets. The Federal University of Ouro Preto contributed to the project providing logistic support and copies of the questionnaire to be distributed to every participating student. All this information is addressed in the the study protocol. All these funders had no role in the design and conduction of this study nor in the preparation, review or approval of this manuscript.		
**X26-i) Comment on ethics committee approval**		
**x26-ii) Outline informed consent procedures**		
**X26-iii) Safety and security procedures**		
**X27-i) State the relation of the study team towards the system being evaluated**		
